# A continuum of HIV care describing mortality and loss to follow-up: a longitudinal cohort study

**DOI:** 10.1016/S2352-3018(18)30048-1

**Published:** 2018-06-04

**Authors:** Sophie Jose, Valerie Delpech, Alison Howarth, Fiona Burns, Teresa Hill, Kholoud Porter, Caroline A Sabin, UK CHIC Study Steering Committee, Jonathan Ainsworth, Sris Allan, Jane Anderson, Abdel Babiker, David R Chadwick, Duncan Churchill, David Dunn, Richard Gilson, Mark Gompels, Phillip Hay, Margaret Johnson, Stephen Kegg, Clifford Leen, Fabiola Martin, Dushyant Mital, Mark Nelson, Chloe Orkin, Adrian Palfreeman, Andrew Phillips, Deenan Pillay, Frank Post, Jillian Pritchard, Achim Scwenk, Anjum Tariq, Roy Trevelion, Andy Ustianowski, John Walsh

**Affiliations:** a Centre for Clinical Research, Epidemiology, Modelling and Evaluation, Institute for Global Health, University College London, London, UK; b Centre for Clinical Research in Infection and Sexual Health, Institute for Global Health, University College London, London, UK; c Public Health England, London, UK; d National Institute for Health Research Health Protection Research Unit in Blood Borne and Sexually Transmitted Infections at University College London, London, UK; e Royal Free London NHS Foundation Trust, London, UK

## Abstract

**Background:**

The cross-sectional HIV care continuum is widely used to assess the success of HIV care programmes among populations of people with HIV and the potential for ongoing transmission. We aimed to investigate whether a longitudinal continuum, which incorporates loss to follow-up and mortality, might provide further insights about the performance of care programmes.

**Methods:**

In this longitudinal cohort study, we included individuals who entered the UK Collaborative HIV Cohort (CHIC) study between Jan 1, 2000, and Dec 31, 2004, and were linked to the national HIV cohort database (HIV and AIDS Reporting System). For each month during a 10 year follow up period, we classified individuals into one of ten distinct categories according to engagement in care, antiretroviral therapy (ART) use, viral suppression, loss to cohort follow-up and loss to care, and mortality, and assessed the proportion of person-months of follow-up spent in each stage of the continuum. 5 year longitudinal continuums were also constructed for three separate cohorts (baseline years of entry 2000–03, 2004–07, and 2008–09) to compare changes over time.

**Findings:**

We included 12 811 people contributing 1 537 320 person-months in our analysis. During 10 years of follow-up, individuals spent 811 057 (52·8%) of 1 537 320 person-months on ART. Of the 811 057 person-months spent on ART, individuals had a viral load of 200 copies per mL or less for 607 185 (74·9%) person-months. 10 years after cohort entry, 3612 (28·1%) of 12 811 individuals were lost to follow-up, 954 (26·4%) of whom had transferred to a non-CHIC UK clinic for care. By 10 years, 759 (5·9%) of 12 811 participants who entered the cohort had died. Loss to follow-up decreased and the proportion of person-months that individuals spent virally suppressed increased over calendar time.

**Interpretation:**

Loss to follow-up in HIV care programmes was high and rates of viral suppression were lower than previously reported. Complementary information provided by a longitudinal continuum might highlight areas for intervention along the HIV care pathway, however, transfers outside the cohort must be accounted for.

**Funding:**

Medical Research Council, UK.

## Introduction

For both the individual and population-wide benefits of antiretroviral therapy (ART) to be realised, people with HIV need to be diagnosed promptly, to engage with HIV care, and to initiate and to adhere to lifelong therapy. The HIV continuum of care has become a widely used approach to describe the benchmark stages along the HIV care pathway to viral suppression. The cross-sectional design of a traditional continuum of care enables up-to-date information about the proportion of people with HIV at each stage of the care pathway to be presented for a specific population at a particular timepoint in one comprehensible figure. Thus, the continuum is useful for monitoring public health and comparing the performance of HIV care programmes, which is particularly relevant in view of public health goals to minimise ongoing transmission and the UNAIDS 90-90-90 target.^1^ However, a longitudinal continuum of care might provide further insights into understanding the success of HIV care programmes. A longitudinal framework has the potential to estimate the time taken for a population to progress from one stage of the continuum to the next, which is an important distinction considering that late HIV diganosis and late ART intitiation have been shown to have a negative effect on rates of transmission and clinical outcomes.^2^, ^3^, ^4^, ^5^, ^6^ An individual's position in the later stages of the continuum might change over time due to loss of virological suppression, treatment interruptions, or disengagement from care. These transitions are not evident in a typical cross-sectional continuum. Furthermore, many individuals with HIV are not accounted for; losses along the care continuum are not well characterised and only individuals who are alive at the time of assessment are included, which is likely to overestimate the spectrum of engagement in care.

The 90-90-90 UNAIDS continuum is under review with suggested expansion to include a so-called fourth 90, which takes into account quality of life.^7^ Other alternative methods of describing a continuum of care include the consideration of a so-called states and transitions framework that additionally describes the rate of transition both forward and backward between stages of the continuum,^8^ time-to-event analyses of the stages of the continuum using longitudinal cohort data,^9^ and the incorporation of mortality as an outcome along the HIV care pathway.^10^, ^11^ However, stages along the continuum are usually considered to be fixed and are assumed not to vary with time. Although Nosyk and colleagues^12^ used a combination of fixed stages and stages that could change over time to present a series of cross-sectional care continuums over time, mortality was not included.

**Research in context**

**Evidence before this study**

We searched PubMed for titles and abstracts of literature published in English between Jan 1, 2011, and Dec 31, 2017, using the search terms “continuum of care”, “care continuum”, “cascade of care”, and “care cascade”. Search results were reviewed to identify publications describing longitudinal or cohort care continuums. National surveillance reports for the UK were also reviewed to obtain current estimates of the continuum of care in the UK. The continuum of care in the UK in 2015, estimated that 83% of individuals with HIV were receiving antiretroviral therapy (ART) and 79% were virologically suppressed. Cross-sectional continuums of care have certain limitations because they do not incorporate patient outcomes or engagement fully, which is crucial for measuring the success of HIV care, and thus methods of presenting a longitudinal HIV continuum of care are starting to be explored. Such cohort continuums might have only a short period of follow-up after HIV diagnosis, and thus only include newly diagnosed individuals. Two studies have additionally incorporated mortality endpoints in the care continuum. One of the studies used cumulative incidence estimates from time-to-event analyses to describe the mean time taken to progress through stages of care. Another study reported the proportion of individuals with optimal, suboptimal, and poor outcomes, including loss to follow-up and death, up to 12 months after enrolment. A states and transitions framework has been theorised, which would describe rates of transition between the stages of HIV care, and the proportion of people at each stage. However, to date, an entire pathway has not been estimated in any setting because extensive data would be required. Although these methods have both advantages and disadvantages, no single method for estimating a longitudinal continuum of care has been recommended.

**Added value of this study**

We have used comprehensive clinical data from a large observational dataset, linked to national cohort and mortality data, to devise a longitudinal measure of the continuum of HIV care. This method uses time-updated factors to account for changes in viral suppression and engagement in care with time, and incorporates loss to care and mortality data, presenting population-level data in a single comprehensible figure. Our study reports the proportion of person-months individuals with HIV spent in suboptimum care categories during a 10 year follow-up period. We found that individuals spent 5% of person-months ART-naive with a CD4 count of 350 cells per μL or less and 25% of person-months on ART with a detectable viral load. Furthermore, 28% of individuals were lost to follow-up 10 years after entry, with a quarter of this loss to follow-up explained by transfer of care.

**Implications of all the available evidence**

A longitudinal continuum of care provides additional insights regarding the success of HIV care programmes for a particular setting, and identifies areas for improvement. Data on cumulative loss to follow-up provides insights into the potential number of people not receiving HIV care, which, if out-migration is low, could contribute substantially to the ongoing epidemic. Early interventions to improve engagement in care are a priority for achieving an optimal continuum of care.

Therefore, the aim of this study was to develop a method to outline the progression of a population of individuals diagnosed with HIV through the stages of the HIV care pathway during a 10 year period, incorporating the outcomes of loss to cohort follow-up and loss to care and mortality.

## Methods

### Study design and participants

In this longitudinal cohort study, we included individuals who entered the UK Collaborative HIV Cohort (CHIC) study^13^ between Jan 1, 2000, and Dec 31, 2004, to allow for a maximum of 10 years of follow-up, ending no later than Dec 31, 2014. The UK CHIC study is an ongoing cohort of HIV-positive individuals (aged >16 years) who have accessed care at 21 HIV clinics in the UK at any time from 1996 onwards, which is linked to the national HIV surveillance cohort based in Public Health England (the HIV and AIDS Reporting System [HARS]). Individuals who were not linked to the HARS were excluded from our analyses, to ensure we could reliably estimate transfer and true loss to care for all individuals.^14^ Mortality data in the UK CHIC Study are reported by participating centres and supplemented through linkage to the HARS, which is linked to the Office for National Statistics mortality registry.^6^, ^14^

We also estimated 5 year continuums, allowing individuals who entered the cohort between Jan 1, 2005, and Dec 31, 2009, to be additionally included.

The UK CHIC Study was approved by the West Midlands multicentre research ethics committee and local ethics committees, and does not require informed consent.

### Procedures

Data was provided electronically by each participating centre annually. Baseline was the date of entry into the study with a follow-up end date that was 10 years after cohort entry, regardless of death or loss to follow-up. For each month during the 10 year follow-up period, we classified individuals into one of ten categories on the basis of current engagement in care, ART use, viral suppression, loss to follow-up, and death to assess the proportion of person-months of follow-up spent in each stage of the care continuum (appendix p 1). We defined engagement in care on a monthly basis using the REACH algorithm.^15^ Months of follow-up were classified as in care if an individual was adhering to a predicted visit schedule, and not in care if an individual was not compliant with a predicted visit schedule. ART use was defined once an individual was reported to have started any antiretroviral drugs. Individuals were considered to have viral suppression if they had a viral load of 200 copies per mL or less, recorded in the previous 9 months. Since individuals stable on ART at the time of this study would be expected to have assessment of viral load every 6 months according to the British HIV Association monitoring guidelines, a 9 month period was chosen to allow some flexibility around the expected timeframe for viral load measurements.^16^ If no viral load was recorded in this period, the viral load was assumed to be detectable.

British HIV Association standards of care recommend all individuals with HIV attend a HIV clinic for care at least once in 12 months, thus loss to follow-up was defined when an individual was classified as not in care for at least 9 consecutive months.^17^ Therefore, individuals predicted to return for treatment within 2 months of an observed visit who were lost to follow-up would not have attended for care for at least 11 months, and individuals predicted to return within 6 months would not have attended for at least 15 months. We further classified person-months lost to follow-up as transfer if individuals were lost to follow-up but had a HARS record of attendance at a non-CHIC centre for that year. Person-months were classified as true loss to care if individuals were lost to follow-up with no HARS record of attendance at a non-CHIC clinic. Once an individual had died, they were categorised as such for all remaining months.

To compare changes across calendar years, we reduced the period of follow-up to 5 years, and estimated the care continuum for three separate cohorts according to year of entry: 2000–03 (to end of 2008), 2004–07 (to end of 2011), 2008–09 (to end of 2014). These periods were selected to correspond with changes in HIV treatment guidelines for initiation of ART in 2008.

### Statistical analysis

We generated and summarised the longitudinal continuum categories and generated stacked area charts using SAS software (version 9.4; SAS Institute, Cary, NC, USA). We estimated the proportion of all person-months of follow-up spent in each state over the duration of the continuum as the number of person-months categorised to each state, divided by the total months of person follow-up in the longitudinal continuum. Cross-sectional assessments at any timepoint relative to cohort entry (eg, 12, 24, 36 months) were the number of person-months in that continuum state at that time, divided by the total number of person-months included at that time (also equal to the number of people included in the continuum for all timepoints). We also did sensitivity analyses to test the definition of loss to cohort follow-up and investigated the inclusion of treatment interruption in the care continuum categories (appendix pp 4, 5).

### Role of the funding source

The funder of the study had no role in study design, data collection, data analysis, data interpretation, or writing of the report. The corresponding author had full access to all the data in the study and had final responsibility for the decision to submit for publication.

## Results

A total of 13 762 individuals were entered into the UK CHIC study between 2000, and 2004, of whom 12 811 (93·1%) were linked to a HARS record. Thus 12 811 individuals with HIV, contributing 1 537 320 person-months, were included in our analyses. Individuals who were linked to a HARS record were more likely to be men (p<0·0001) and to have acquired HIV through sex between men or through sex between men and women (p<0·0001) than individuals who were not linked (table). Individuals who were not linked to a HARS record were more likely to have missing data on ethnicity (179 [18·8%] of 951 individuals *vs* 423 [3·3%] of 12 811 individuals) and route of HIV acquisition (256 [26·9%] of 951 individuals *vs* 871 [6·8%] of 12 811 individuals) than individuals linked to a HARS record (p<0·0001). A higher proportion of individuals not linked to a HARS record were lost to follow-up 10 years after cohort entry than individuals linked to a HARS record (453 [47·6%] of 951 individuals *vs* 3613 [28·2%] of 12 811 individuals).

**Table:** Characteristics of individuals enrolled in the UK CHIC study by year of cohort entry

		**Not linked to HARS (2000–04 [n=951])**	**10 year cohort (2000–04 [n=12 811])**	**5 year cohorts**		
				2000–03 (n=9954)	2004–07 (n=11 259)	2008–09 (n=5318)
Age (years)						
	<30	283 (29·8%)	4004 (31·3%)	3182 (32·0%)	3344 (29·7%)	1414 (26·6%)
	30–39	409 (43·0%)	5698 (44·5%)	4458 (44·8%)	4669 (41·5%)	2030 (38·2%)
	40–49	165 (17·4%)	2239 (17·5%)	1650 (16·6%)	2379 (21·1%)	1324 (24·9%)
	≥50	82 (8·6%)	870 (6·8%)	664 (6·7%)	867 (7·7%)	550 (10·3%)
Sex						
	Men	567 (59·6%)	8396 (65·5%)	6565 (66·0%)	7416 (65·9%)	3686 (69·3%)
	Women	373 (39·2%)	4415 (34·5%)	3389 (34·0%)	3843 (34·1%)	1632 (30·7%)
Ethnicity						
	White	344 (36·2%)	5646 (44·1%)	4399 (44·2%)	5106 (45·4%)	2543 (47·8%)
	Black	341 (35·9%)	5660 (44·2%)	4334 (43·5%)	4914 (43·6%)	2087 (39·2%)
	Other or unknown	266 (28·0%)	1505 (11·7%)	1221 (12·3%)	1239 (11·0%)	688 (12·9%)
Mode of HIV acquisition						
	Sex between men	257 (27·0%)	5157 (40·3%)	4054 (40·7%)	4617 (41·0%)	2205 (41·5%)
	Heterosexual	396 (41·6%)	6343 (49·5%)	4873 (49·0%)	5477 (48·6%)	2355 (44·3%)
	Other or unknown	298 (31·3%)	1311 (10·2%)	1027 (10·3%)	1165 (10·3%)	758 (14·3%)
Newly diagnosed		723 (76·0%)	9888 (77·2%)	7948 (79·8%)	8110 (72·0%)	3737 (70·3%)
CD4 count (cells per μL)						
	<200	142 (14·9%)	2838 (22·2%)	2226 (22·4%)	2582 (22·9%)	1115 (21·0%)
	201–350	83 (8·7%)	1998 (15·6%)	1463 (14·7%)	2101 (18·7%)	1057 (19·9%)
	>350	199 (20·9%)	3414 (26·6%)	2509 (25·2%)	3833 (34·0%)	2081 (39·1%)
	Data not available	527 (55·4%)	4561 (35·6%)	3756 (37·7%)	2743 (24·4%)	1065 (20·0%)
Viral load (log_10_copies per mL)		4·2 (2·6–5·0)	4·3 (3·1–5·1)	4·4 (3·3–5·1)	4·2 (2·8–5·0)	4·0 (2·5–4·9)

Data are n (%), or median (IQR). CHIC=Collaborative HIV Cohort. HARS=HIV and AIDS Reporting System.

The mean age at study entry for the 10-year cohort was 34 years (SD 9·2). Ethnicity was balanced across the included cohort (table). The proportion of individuals older than 50 years, and the number of individuals with CD4 counts higher than 350 cells per μL at baseline increased over time (table).

**Figure 1:**
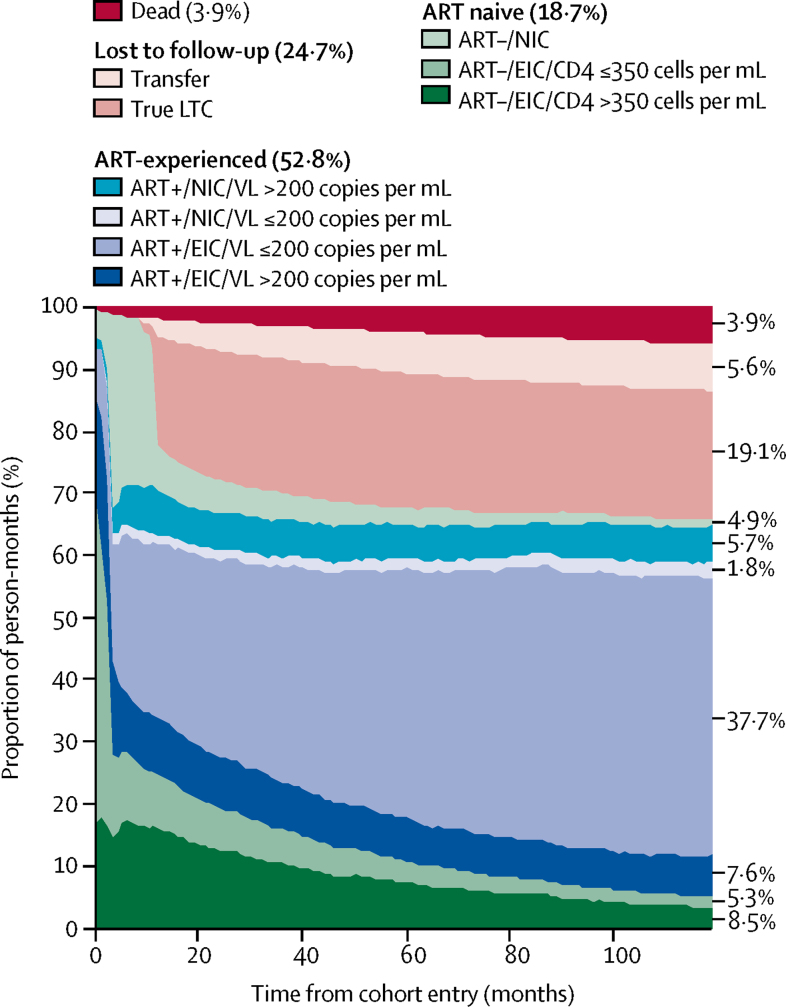
10 year longitudinal continuum of care Person-months for each stage of the continuum: ART–/EIC/CD4 >350, 130 114 person-months; ART–/EIC/CD4 ≤350, 82  102 person-months; ART–/NIC, 74 917 person-months; ART+/EIC/VL >200, 116 599 person-months; ART+/EIC/VL ≤200, 579 410 person-months; ART+/NIC/VL ≤200, 27 775 person-months; ART+/NIC/VL >200, 87 273 person-months; true LTC, 293 048 person-months; transfer, 86 771 person-months; and dead, 59 311 person-months. See appendix (p 1) for a full description of each stage of the continuum. LTC=loss to care. ART+=antiretroviral therapy experienced. ART–=antiretroviral therapy naive. NIC=not in care. EIC=engaged in care. VL=viral load. ART–=antiretroviral therapy naive.

The 10 year longitudinal continuum of care included a total 1 537   320 person-months of follow-up (figure 1), of which individuals spent 811 057 (52·8%) person-months on ART. Overall, individuals had viral loads of 200 copies per mL or less for 607 185 (39·5%) of 1 537 320 person-months, which accounted for 607 185 (74·9%) of 811 057 person-months spent on ART. Of 1 098 190 person-months spent alive and retained in the cohort, individuals were ART-experienced for 811 057 (73·9%) person-months and were virologically suppressed for 607 185 (55·3%) person-months.

A total of 287 133 person-months were spent ART-naive, of which 130 114 (45·3%) were spent engaged in care with a CD4 count higher than 350 cells per μL, 82 102 (28·6%) person-months were spent engaged in care with a CD4 count of 350 cells per μL or less, and 74 917 (26·1%) person-months were categorised as not in care. Of 82 102 person-months spent ART-naive with a CD4 count of 350 cells per μL or less, only 10 674 (13·0%) of these person-months corresponded to the 3 months following HIV diagnosis. The number of person-months spent engaged in care was higher for participants who had initiated ART than participants who were ART-naive, with 115 048 (14·2%) of 811 057 person-months spent ART-experienced classified as not in care, compared with 74 917 (26·1%) of 287 133 person-months spent ART-naive. A total of 379 819 (24·7%) of 1 537 320 person-months were classified as lost to follow-up, of which 86 771 (22·8%) of 379 819 person-months were categorised as transfer to another HIV clinic for care. Thus, 293 048 (19·1%) of 1 537 320 person-months represented true loss to care (figure 1). In total, 759 (5·9%) of 12 811 individuals had died within 10 years of cohort entry, representing 59 311 (3·9%) of 1 537 320 person-months lost due to death (figure 1).

The number of individuals receiving ART increased with time from entry (3304 [25·8%] of 12 811 individuals at entry *vs* 7676 (59·9%) of 12 811 individuals 10 years after entry; appendix p 2). However, 764 (6·0%) of 12 811 individuals were retained, but had not received ART 10 years after entry into care. 1036 (31·4%) of 3304 of individuals who were receiving ART at entry had a viral load of 200 copies per mL or less (8·0% of all individuals), and 6029 (78·5%) of 7676 individuals who had received ART and were in follow-up at 10 years had a viral load of 200 copies per mL or less. Thus, 6029 (47·0%) of 12 811 individuals in the cohort had a viral load of 200 copies per mL or less 10 years after cohort entry. The proportion of individuals lost to follow-up increased rapidly in the 2 years after entry, and 10 years after cohort entry, 3612 (28·1%) of 12 811 individuals were lost to follow-up, of whom 954 (26·4%) had transferred to a non-CHIC UK clinic for care (appendix p 2). The number of patients classified as true loss to care remained constant across the cohort between 2 years and 10 years after entry (2650 [20·7%) of 12 811 individuals *vs* 2568 [20·7%] of 12 811 individuals), whereas loss to follow-up due to transfer increased between 2 years and 10 years after entry (556 [4·3%] *vs* 954 [7·4%]; appendix p 2). As a proportion of all individuals lost to cohort follow-up, transfer increased from 17·2% (556 of 3206 individuals) at 2 years after cohort entry to 26·4% (954 of 3612 individuals) at 10 years after cohort entry (appendix p 2).

**Figure 2:**
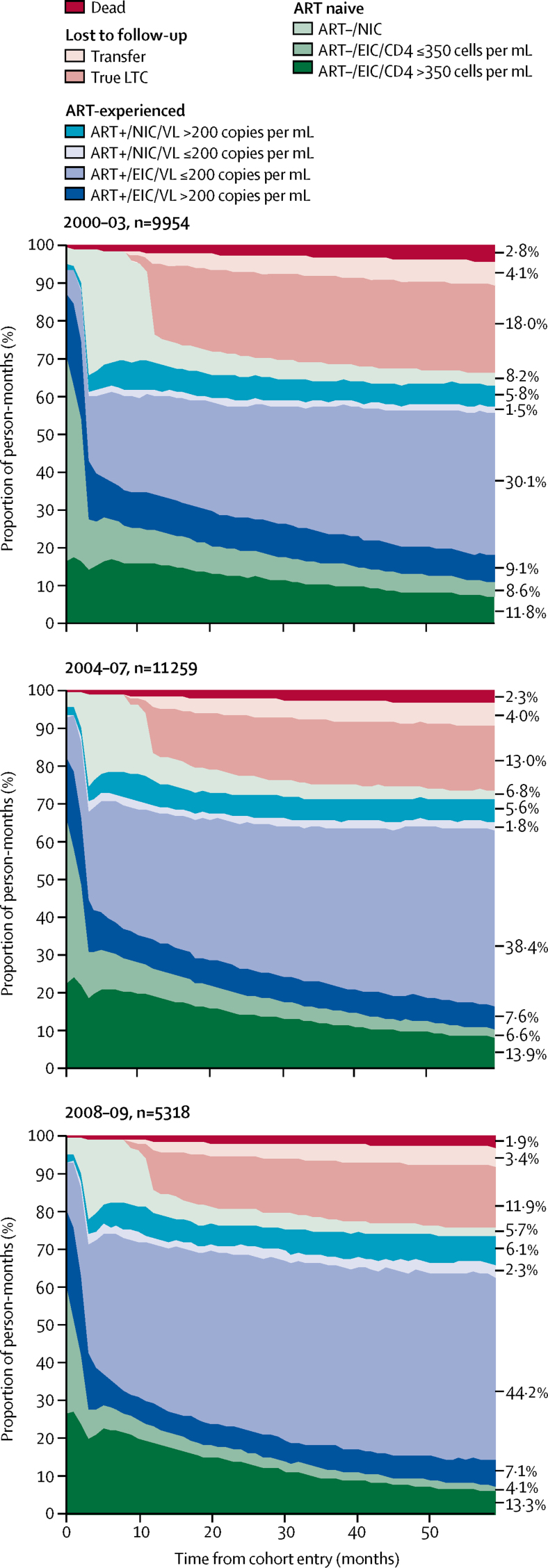
5 year longitudinal care continuum by calendar year of cohort entry Person-months for each stage of the continuum: ART–/EIC/CD4 >350, 70 576 (2000–03), 93 695 (2004–07), and 42 573 (2008–09) person-months; ART–/EIC/CD4 ≤350, 51 350 (2000–03), 44 498 (2004–07), 13 210 (2008–09) person-months; ART–/NIC, 49 105 (2000–03), 45 749 (2004–07), 18 293 (2008–09) person-months; ART+/EIC/VL >200, 54 154 (2000–03), 51 058 (2004–07), 22 545 (2008–09) person-months; ART+/EIC/VL ≤200, 179 775 (2000–03), 259 598 (2004–07), 140 887 (2008–09) person-months; ART+/NIC/VL ≤200, 8775 (2000–03), 12 408 (2004–07), 7453 (2008–09) person-months; ART+/NIC/VL >200, 34 444 (2000–03), 38 018 (2004–07), 19 395 (2008–09) person-months; true LTC, 107 733 (2000–03), 87 975 (2004–07), 37 805 (2008–09) person-months; transfer, 24 724 (2000–03), 27 032 (2004–07), 10 713 (2008–09) person-months; dead, 16 604 (2000–03), 15 509 (2004–07), 6206 (2008–09) person-months. See appendix (p 1) for a full description of each stage of the continuum. LTC=loss to care. ART+=antiretroviral therapy experienced. ART–=antiretroviral therapy naive. NIC=not in care. EIC=engaged in care. VL=viral load.

We included 9954 individuals (contributing 597 240 person-months) in the 2000–03 cohort, 11 259 individuals (contributing 675 540 person-months) in the 2004–07 cohort, and 5318 individuals (contributing 319 080 person-months) in the 2008–09 cohort. True loss to care was lower in the cohort that entered the study in 2008–09 than the 2000–03 cohort (figure 2). Of the 5318 individuals included in the 2008–09 cohort, 732 (13·8%) individuals were defined as true loss to care 2 years after entry, and 865 (16·3%) individuals at 5 years after entry (appendix p 3). Comparison of the 9954 individuals included in the 2000–03 cohort revealed that 2 years after entry, 2190 (22·0%) individuals were defined as true loss to care, and 2295 (23·1%) of individuals 5 years after entry. The number of participants not in care before ART initiation was also lower in the year after cohort entry in the 2008–09 cohort than the 2000–03 cohort (689 [13·0%] of 5318 individuals *vs* 2307 [23·2%] of 9954 individuals; appendix p 3).

Participants in the 2008–09 cohort spent a lower proportion of person-months before ART initiation engaged in care with a CD4 count of 350 cells per μL or less than did participants in the 2000–03 and 2004–07 cohorts (figure 2). In the 2008–09 cohort, 13 210 (17·8%) of 74 076 person-months before ART initiation were spent engaged in care with a CD4 count of 350 cells per μL or less, compared with 51 350 (30·0%) of 171 031 person-months in the 2000–03 cohort, and 44 498 (24·2%) of 183 942 person-months in the 2004–07 cohort. ART initiation seemed to be more rapid in the most recent cohort than the earlier cohorts; 3036 (57·1%) of 5318 individuals had started ART 1 year after entry in the 2008–09 cohort compared with 4446 (44·7%) of 9954 individuals in the 2000–03 cohort and 5662 (50·3%) of 11 259 individuals in the 2004–07 cohort (appendix p 3). The proportion of person-months on ART spent with viral suppression over a 5 year period also increased over time: 188 550 (68·0%) of 277 148 person-months in the 2000–03 cohort, 272 006 (75·3%) of 361 082 person-months in the 2004–07 cohort, and 148 340 (78·0%) of 190 280 person-months in the 2008–09 cohort (figure 2). Mortality seemed to be moderately lower in the 2008–09 cohort than the 2000–03 cohort (figure 2).

Sensitivity analyses showed that the length of time used to define loss to follow-up did not change the rate of loss to follow-up substantially, and inclusion of treatment interruption in the care continuum did not alter our findings substantially (appendix pp 4,5).

## Discussion

We observed an increasing probability of ART use and subsequent viral suppression with time from entry into HIV care in this longitudinal continuum of care. However, 10 years after study entry, 6·0% of individuals were alive and accessing care (ie, not lost to follow-up), but had not started ART, 12·9% were accessing care and had initiated ART, but had not achieved viral suppression, and 20·7% were lost to care.

We present a novel method of describing population-level progress through the stages of the care pathway within the HIV continuum of care. By establishing categories to represent stages along the care continuum, and suboptimum responses along the care pathway, all individuals who enter care are accounted for. Following a closed population for a fixed number of years enabled us to incorporate loss to follow-up and mortality data, providing additional insights into the traditional continuum of care. We used time-updated measures of the stages of the care pathway if possible, allowing individuals to move between stages while maintaining a population-based overview.

In this study, participants spent 74·9% of person-months on ART with a viral load below 200 copies per mL. This result indicates worse virological outcomes among people living with HIV than that of a traditional cross-sectional continuum of care in the UK, which reports viral suppression rates of 94% in people given ART.^18^ Although these estimates are not directly comparable, they highlight the different conclusions that might be drawn from a cross-sectional versus longitudinal continuum of care.^9^, ^19^ The proportion of person-months spent with an unsuppressed viral load has implications for both ongoing transmission and individual outcomes, and is therefore a useful measure of programmatic success. The context in which this non-suppression occurs is also important; if viral suppression is not achieved or lost due to poor adherence during periods of high-risk behaviours, this is of particular concern in view of the potential for onward transmission.

Loss to care was higher in this longitudinal continuum of care than that previously reported using cross-sectional measures. National surveillance data estimate that 95% of individuals who attend HIV clinics for care in a calendar year will return for care the following year.^20^ The increasing cumulative rate of loss to care in this study is consistent with previous reports that one in five people who attend for care in the UK will be lost to follow-up within 5 years,^21^, ^22^ and suggests that a substantial group of patients disengage from care for periods of time and are slow to, or never, re-engage. Most individuals who were lost to follow-up in this study were lost to follow-up within 2 years of entry to care, suggesting that interventions to improve engagement soon after diagnosis are needed. A previous study^23^ in the UK found newly diagnosed individuals, women, younger adults, black African individuals, and individuals who have acquired HIV outside of the UK are most likely to be lost to follow-up. In our analysis only around a quarter of loss to follow-up was explained by transfer of care elsewhere, with the remainder unaccounted for. Some of this loss to care might be explained by migration; in the UK, approximately a quarter of individuals who do not return for care and who can be traced are thought to have left the country, but most people cannot be accounted for.^20^, ^23^ Therefore, for most individuals lost to care who remain in the country, but do not access care, this might have important consequences for individual and public health, since individuals who are disengaged from care are more likely to transmit HIV to others and to have poor health outcomes themselves, possibly only re-engaging with care when they become ill.^2^, ^3^ This underscores the importance of ensuring that all people with HIV have access to HIV treatment, free at the point of use and, although we have no information on the migration status of individuals lost to care, such access needs to include documented and undocumented migrants, which is the standard procedure in the UK.

Changes were observed in the longitudinal continuum of care over time. Fewer person-months were spent naive to ART with CD4 counts of 350 cells per μL or less, ART initiation was more rapid, more individuals achieved viral suppression, and fewer individuals were lost over time. These differences are likely to reflect changes in treatment guidelines and developing knowledge about the benefits of ART initiation at high CD4 cell counts during this time period.^24^ Thus, although ART initiation was less rapid in the earlier cohorts than the later cohorts, this might simply reflect adherence to treatment guidelines at the time. Earlier ART initiation in the cohort who entered the study between 2008, and 2009, might contribute to the lower number of person-months categorised as lost to follow-up over time. A lower proportion of person-months after ART initiation were classified as not in care than before ART initiation, consistent with other studies, albeit in different health-care settings.^25^, ^26^, ^27^ Whether accumulation of follow-up data in the setting of immediate initiation of ART will result in lower overall loss to care should be monitored.

This approach is similar to that of McNairy and colleagues,^19^ who categorised the outcomes of all individuals who entered care into poor, suboptimal, or optimal responses at several points during the first year of ART. The long follow-up of the present study, and more detailed categorisation of the stages of the care pathway, provides further insights about the long-term experience of HIV care in the UK. In our analysis, we include time-updated measures of both viral suppression and engagement in care that are more reflective of real-life scenarios in which individuals can both disengage from and re-engage with care and rebound and suppress their viral load. The use of the large clinical cohort from the UK CHIC Study, which represents more than half of all people diagnosed with HIV in the UK, and the availability of a national cohort to which it can be linked, is a strength of this study. This linkage allows us to reliably account for transfer of care and assess true loss to care rates and mortality. Smaller clinical cohorts not linked to national HIV care data might generate inflated estimates of loss to follow-up, particularly if people frequently transfer care between HIV clinics.

Our study has limitations. Our study followed up a closed cohort over a long period of time, thus our findings might be less generalisable to more recent cohorts with different demographic and social characteristics and for whom treatment guidelines have changed. We have included a selected population of individuals linked to care rather than a newly diagnosed population, and of those, only individuals who could be linked to a HARS record. In the UK, 97% of newly diagnosed individuals link to care within 3 months. This rate of linkage is similar across age, sex, ethnicity, and HIV risk subgroups, which would indicate that the population who link to care are largely representative of the whole diagnosed population.^28^ In the present study, individuals linked to a HARS record were more likely to have available data on ethnicity and route of HIV acquisition, and were less likely to be lost to follow-up than individuals not linked to HARS. These differences could indicate that individuals who could not be linked to a HARS record are less likely to be engaged in care or are more likely to transfer between HIV care centres than individuals linked to HARS, which could result in some bias in our estimated rates of transfer and loss to care. Because our study investigated a cohort linked to HIV care, we have not measured all stages of the continuum of care. This method could not be easily applied to the first stage of the continuum (ie, to estimate person-time spent undiagnosed) since reliable dates of HIV acquisition are rarely known. The absence of data on out-migration presents a challenge in truly understanding how much of this loss to care is explained by people who remain in the country without accessing care.

Although a cross-sectional continuum of care is a useful tool for assessing the potential public health impact of programmatic performance, additional insights can be gained from a longitudinal approach. Understanding patterns and determinants of both disengagement and re-engagement with care and the role of migration is important for improving retention of individuals within the HIV care pathway in the UK.
