# Supplementary material for: A continuum of HIV care describing mortality and loss to follow-up: a longitudinal cohort study
Source: Lancet HIV. 2018 Jun 4;5(6):e301–8. doi: 10.1016/S2352-3018(18)30048-1 (PMC5990495; doi:10.1016/S2352-3018(18)30048-1)
Supplement: Supplementary appendix [file mmc1.pdf]

THE LANCET
HIV

Supplementary appendix

This appendix formed part of the original submission and has been peer reviewed. We post it as supplied by the authors.

Supplement to: Jose S, Delpech V, Howarth A, et al, on behalf of the UK CHIC Study Steering Committee. A continuum of HIV care describing mortality and loss to follow-up: a longitudinal cohort study. *Lancet HIV* 2018; **5:** e301–08.

Supplementary Table 1: Stages of the time-updated Continuum of care

Stage definition	Notation
**ART Naïve:**	
Engaged in Care<br>CD4 >350 cells per µL<br>VL >200 copies per mL or missing	ART – / EIC / CD4 >350
Engaged in Care<br>CD4 ≤350 cells per µL or CD4 unavailable<br>VL >200 copies per mL or missing	ART – / EIC / CD4 <350
Not in care<br>VL >200 copies per mL or missing	ART – / NIC
**ART-experienced:**	
Engaged in care<br>VL >200 copies per mL or missing	ART + / EIC / VL >200
Not in care<br>VL >200 copies per mL or missing	ART + / NIC / VL >200
Engaged in care<br>VL ≤200 copies per mL	ART + / EIC / VL ≤200
Not in care<br>VL ≤200 copies per mL	ART + / NIC / VL ≤200
**Lost to cohort follow-up (LTCFU):**	
True loss to care - cumulative non-attendance for >9 months with no transfer outside the cohort	True LTC
Transfer - seen for care outside the cohort during calendar year of LTCFU	Transfer
**Died**	Dead

EIC= Engaged in care; NIC= Not in care; ART+ = Started ART; ART- = Not started ART; VL=Viral load.

Supplementary Table 2: Percentage of individuals in each stage of the longitudinal care continuum according to time from cohort entry

Percentage (n) of individuals in each stage of continuum	Months from cohort entry					
	1	24	48	72	96	120
**ART-Naïve:**						
**ART – / EIC / CD4 >350**	17·0 (2174)	12·7 (1627)	8·5 (1086)	6·3 (801)	4·4 (566)	3·3 (412)
**ART – / EIC / CD4 <350**	52·5 (6722)	6·9 (880)	4·4 (567)	2·9 (376)	2·1 (268)	1·6 (207)
**ART – / NIC**	4·3 (552)	5·6 (712)	3·8 (481)	2·4 (313)	1·7 (212)	1·1 (138)
**ART-Experienced:**						
**ART + / EIC / VL >200**	16·0 (2046)	8·4 (1075)	7·3 (936)	6·7 (856)	6·2 (795)	6·9 (880)
**ART + / EIC / VL ≤200**	7·8 (995)	31·5 (4035)	37·2 (4764)	41·6 (5326)	44·5 (5703)	44·5 (5703)
**ART + / NIC / VL ≤200**	0·3 (41)	1·5 (198)	1·6 (201)	1·8 (235)	2·1 (272)	2·5 (326)
**ART + / NIC / VL >200**	1·7 (222)	5·9 (750)	5·9 (756)	5·5 (705)	5·8 (749)	6·0 (767)
**Lost to cohort follow-up:**						
**True LTC**	0·0 (0)	20·7 (2650)	21·9 (2809)	21·5 (2752)	20·7 (2655)	20·7 (2658)
**Transfer**	0·0 (0)	4·3 (556)	5·9 (756)	6·8 (872)	7·2 (919)	7·4 (954)
**Dead**	0·5 (59)	2·6 (328)	3·5 (449)	4·5 (575)	5·2 (672)	5·9 (759)

EIC=Engaged in care; NIC=Not in care; ART +=ART-experienced; ART-=ART-Naïve; VL=viral load; LTC=Loss to care

Supplementary Table 3: Percentage of individuals in each stage of the longitudinal care continuum according to time from cohort entry, according to calendar year of cohort entry

Proportion (n) of individuals in each stage of continuum	2000-03 (N=9954)					2004-07 (N=11259)					2008-09 (N=5318)				
	Months from cohort entry					Months from cohort entry					Months from cohort entry				
	12	24	36	48	60	12	24	36	48	60	12	24	36	48	60
**ART-Naïve:**															
**ART - / EIC / CD4 >350**	16·1 (1602)	12·5 (1249)	10·4 (1038)	8·4 (834)	7·3 (725)	19·3 (2169)	14·6 (1640)	11·8 (1330)	9·7 (1095)	7·8 (879)	19·4 (1034)	14·2 (754)	10·0 (532)	7·9 (421)	6·3 (333)
**ART - / EIC / CD4 <350**	8·9 (889)	7·1 (708)	5·7 (563)	4·6 (462)	3·7 (365)	7·9 (887)	5·6 (625)	4·0 (446)	2·8 (320)	2·1 (241)	4·8 (253)	3·1 (164)	2·4 (129)	2·0 (105)	1·5 (80)
**ART - / NIC**	23·2 (2307)	5·6 (556)	4·6 (453)	3·9 (390)	3·0 (300)	16·5 (1861)	5·5 (616)	4·0 (448)	3·0 (340)	2·4 (274)	13·0 (689)	4·5 (237)	3·7 (198)	2·6 (138)	2·2 (116)
**ART-Experienced:**															
**ART + / EIC / VL >200**	9·6 (953)	8·6 (857)	8·3 (829)	7·6 (757)	7·2 (719)	7·1 (799)	7·0 (785)	6·8 (765)	6·5 (736)	6·2 (701)	6·0 (320)	6·0 (321)	6·4 (338)	5·8 (306)	6·9 (368)
**ART + / EIC / VL ≤200**	26·0 (2587)	29·3 (2914)	33·0 (3289)	35·6 (3542)	37·9 (3771)	34·1 (3836)	38·1 (4288)	41·3 (4649)	44·8 (5040)	46·9 (5277)	41·6 (2214)	45·5 (2419)	47·7 (2535)	48·1 (2557)	48·0 (2551)
**ART + / NIC / VL ≤200**	1·3 (131)	1·7 (169)	1·4 (140)	1·6 (156)	1·5 (152)	1·7 (189)	1·6 (181)	1·7 (197)	1·8 (199)	1·8 (207)	2·0 (104)	2·1 (110)	2·0 (109)	3·3 (177)	3·4 (181)
**ART + / NIC / VL >200**	8·0 (795)	6·3 (624)	5·5 (547)	5·6 (556)	5·5 (551)	7·4 (838)	5·5 (616)	5·7 (641)	5·8 (652)	6·1 (688)	7·5 (398)	5·5 (290)	6·2 (328)	7·1 (376)	7·6 (403)
**Lost to cohort follow-up:**															
**True LTC**	3·7 (372)	22·0 (2190)	22·8 (2274)	23·0 (2291)	23·1 (2295)	3·3 (377)	15·7 (1764)	16·8 (1894)	17·0 (1910)	17·0 (1914)	3·6 (191)	13·8 (732)	15·1 (803)	15·9 (844)	16·3 (865)
**Transfer**	1·3 (129)	4·3 (426)	5·2 (518)	6·0 (600)	6·6 (653)	1·2 (135)	4·4 (491)	5·3 (592)	5·5 (624)	6·1 (688)	0·9 (50)	3·5 (188)	4·3 (231)	4·9 (260)	4·9 (262)
**Dead**	1·9 (189)	2·6 (261)	3·0 (303)	3·7 (366)	4·2 (423)	1·5 (168)	2·2 (250)	2·6 (297)	3·0 (343)	3·5 (392)	1·2 (65)	1·9 (103)	2·2 (115)	2·5 (134)	3·0 (159)
EIC=Engaged in care; NIC=Not in care; ART +=ART-experienced; ART-=ART-Naïve; VL=viral load; LTC=Loss to care															

Sensitivity Analyses

Methods

We tested the definition of ART use by considering treatment interruptions, classifying any person-month after ART initiation in which an individual did not appear to have been prescribed ART as a treatment interruption. As this measure relies on treatment stop dates recorded in patient health records, this approach will likely capture only scheduled treatment interruptions, as opposed to interruptions due non-adherence, for example, of which the care-giving clinician may not be aware. We also tested the definition of LTCFU by changing the number of cumulative months required to be NIC to either six or twelve. Finally, we re-estimated the ten-year longitudinal continuum restricting to individuals who had at least two visit dates in the UK CHIC cohort. This is more consistent with previous analyses of the REACH algorithm [1,2] and excludes attenders who have only one record of contact with a clinic, who would become almost immediately LTCFU and are less likely to have complete information available in the UK CHIC Study on demographic or clinical characteristics and outcomes.

Results

Scheduled treatment interruption accounted for a relatively low percentage of person-months over ten years (4·7%; n=72,200)[Supplementary Figure 1]. Discounting those undergoing a treatment interruption, we observed that of all person-months on ART over ten years (n=738,857), 80·1% (n=592,635) were spent virologically suppressed (as opposed to 74.5% in the main analysis).

Using six or twelve months NIC to define LTCFU made little difference to the longitudinal continuum. Using a six month definition we saw that 20·9% (n=321,153) of all person-months over ten years were classified as true LTC and 6·0% (n=92,729) as transfer of care. Using a twelve month definition we observed that 17·6% (n=270,470) and 5·3% (n=81,571) were classified as true LTC and transfer of care. Restricting to 11,272 (88·0%) individuals who had at least two separate visits in the UK CHIC study, rates of true LTC were lower than observed in the main analysis (14.9% (n=201,282)), and a larger proportion of person-months were spent with viral suppression (44·9% (n=606,647)).

References

1. Howarth AR, Burns FM, Apea V, Jose S, Hill T, Delpech VC, et al. Development and application of a new measure of engagement in out-patient HIV care. HIV medicine. 2016.

2. Sabin CA, Howarth A, Jose S, Hill T, Apea V, Morris S, et al. Association between engagement in-care and mortality in HIV-positive persons. AIDS (London, England). 2017;31(5):653-60.

Supplementary Figure 1: Longitudinal continuum of care over 10 years considering treatment interruption

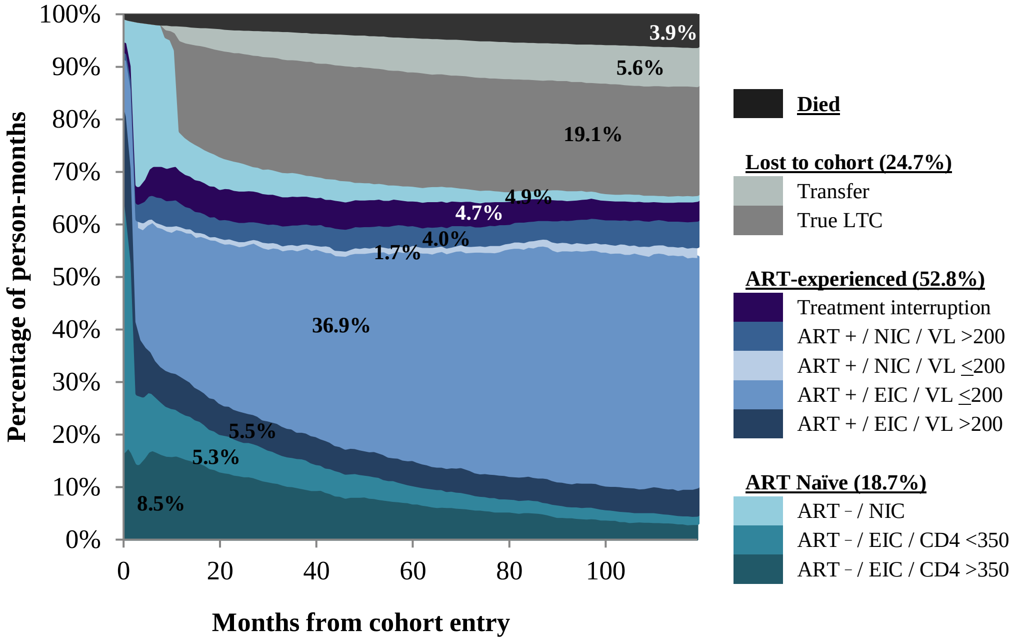

Percentage (n) of individuals in each stage of continuum	Months from cohort entry					
	1	24	48	72	96	120
**ART-Naïve:**						
**ART - / EIC / CD4 >350**	17·0 (2174)	12·7 (1627)	8·5 (1086)	6·3 (801)	4·4 (566)	3·3 (412)
**ART - / EIC / CD4 <350**	52·5 (6722)	6·9 (880)	4·4 (567)	2·9 (376)	2·1 (268)	1·6 (207)
**ART - / NIC**	4·3 (552)	5·6 (712)	3·8 (481)	2·4 (313)	1·7 (212)	1·1 (138)
**ART-Experienced:**						
**ART + / EIC / VL >200**	14.6 (1873)	5.6 (716)	4.9 (630)	4·7 (597)	4.6 (592)	5.3 (683)
**ART + / EIC / VL ≤200**	7·5 (965)	30.8 (3945)	36.4 (4661)	40.7 (5212)	43.7 (5593)	43.4 (5554)
**ART + / NIC / VL ≤200**	0·3 (40)	1·4 (180)	1·4 (185)	1·7 (221)	2·0 (260)	2·4 (309)
**ART + / NIC / VL >200**	1·5 (191)	3.6 (462)	4.1 (521)	3.8 (492)	4.6 (589)	5·0 (645)
**Treatment interruption**	1.8 (235)	5.9 (755)	5.2 (660)	4.7 (600)	3.8 (485)	3.8 (485)
**Lost to cohort follow-up:**						
**True LTC**	0·0 (0)	20·7 (2650)	21·9 (2809)	21·5 (2752)	20·7 (2655)	20·7 (2658)
**Transfer**	0·0 (0)	4·3 (556)	5·9 (756)	6·8 (872)	7·2 (919)	7·4 (954)
**Dead**	0·5 (59)	2·6 (328)	3·5 (449)	4·5 (575)	5·2 (672)	5·9 (759)

ART-/EIC/CD4 >350=ART-Naïve, Engaged in care, CD4 >350 cells per µL (n=130114) ; ART-/EIC/CD4 <350=ART-Naïve, Engaged in care, CD4 <350 cells per µL (n=82102); ART-/NIC=ART-Naïve, Not in care (n=74917); ART+/EIC/VL>200=ART-experienced, Engaged in care, viral load >200 copies per mL (n=84100); ART+/EIC/VL ≤200=ART-experienced, Engaged in care, viral load ≤200 copies per mL (n=567036); ART+/NIC/VL ≤200=ART-experienced, Not in care, viral load ≤200 copies per mL (n=25599); ART+/NIC/VL>200=ART-experienced, Not in care, viral load >200 copies per mL (n=62122); Treatment Interruption (n=72200); True LTC=Lost to cohort follow-up (LTCFU) with no record of attendance in the HIV/AIDS Reporting System (HARS) (n=293048); Transfer=LTCFU with record of attendance in HARS (n=86771).
